# Thinking Aloud: Stress and Coping in Junior Cricket Batsmen During Challenge and Threat States

**DOI:** 10.1177/0031512520938911

**Published:** 2020-07-08

**Authors:** Michael McGreary, Martin Eubank, Robert Morris, Amy Whitehead

**Affiliations:** 1 School of Sport and Exercise Sciences, 4589 Liverpool John Moores University

**Keywords:** concurrent verbalizations, stress, coping, cricket, think-aloud

## Abstract

The present study examined stress and coping of cricket batsmen during challenge and threat states using the Think-Aloud method. Ten male elite-level junior cricket batsmen took part in the study. A repeated measures design was implemented, with participants verbalizing while both in (a) a threat state and (b) a challenge state. Participants were required to score 36 runs in 30 balls during the threat and challenge conditions. Verbalizations were subsequently transcribed verbatim and analyzed for stressors, coping strategies, and any other reoccurring themes. A paired-samples t-test was conducted to examine differences in the number of verbalizations made for each theme between conditions. Ten secondary themes were grouped into four primary themes; these included (a) stressors, (b) problem-focused coping, (c) emotion-focused coping, and (d) gathering information. There were significant differences( p⩽0.05) between stressor verbalizations, with significantly more verbalizations made by participants during a threat state. No significant differences were found between any other themes. Thus, during a threat state, participants reported significantly more stressor verbalizations compared to a challenge state, while there were no significant differences in coping strategies reported (p>0.05). This finding offers a potential explanation for why athletic performance diminishes when in a threat state, as athletes then experience a greater number of stressors but do not report engaging in more coping strategies.

## Introduction

When performing in pressurized environments, athletes commonly experience stress before, during, and sometimes after the event (Moore et al., 2013). Given this, sport psychology researchers have investigated both physiological responses (e.g., Turner et al., 2013) and psychological responses (e.g., Swann et al., 2017) to stress and how these impact on sport performance. It has been argued that stress is a dynamic and recursive transaction between the demands of a situation and an individual’s resources to manage those demands (Lazarus, 1991). Whereas coping has been defined as “constantly changing cognitive and behavioral efforts to manage specific external and/or internal demands that are appraised as taxing or exceeding the resources of the person” (Lazarus & Folkman, 1984, p.141). One theoretical model that has attempted to make sense of individual differences in stress responses is the biopsychosocial model (BPSM) of challenge and threat (Blascovich, 2008). Previously, research has used this model to examine the impact of challenge and threat (CAT) states on the performance of a sporting task (e.g., Moore et al., 2013). Similar to this, the Theory of Challenge and Threat States in Athletes (TCTSA; Jones et al., 2009), which is underpinned by the BPSM, collates physiological and emotional factors underpinning sporting performance. Finally, the Evaluative Space Approach to Challenge and Threat (ESACT; Uphill et al., 2019) was prompted by both the BPSM and TCTSA and argued that individuals could be both challenged and threatened.

The BPSM is underpinned by Lazarus and Folkman’s (1984) transactional theory of stress, and Dienstbier’s (1989) theory of physiological toughness. BPSM proposes that the responses of individuals in motivated situations, such as that of a sporting event, are determined by an individual’s evaluations of the demands of the situation and their resources to cope with them. According to the BPSM, individuals in a challenge state have evaluated that they have the necessary coping resources to match or exceed situational demands. A challenge state is characterised by an increase in heart rate (HR) and cardiac output (CO) and a decrease in total peripheral resistance (TPR). Individuals may enter the threat state when they evaluate the demands of the situation as being greater than their available resources. Much like the challenge state, sympathetic adrenal medullary activation has been hypothesized. However, pituitary-adrenal cortical activation has also been predicted. This activation results in cortisol release, constriction of blood vessels and inhibited effects of sympathetic adrenomedullary activation (Blascovich & Mendes, 2000; Jamieson et al., 2013). According to ESACT (Uphill et al., 2019) challenge and threat are not opposite ends of a bipolar continuum but rather, a unidimensional continuum and as such, individuals can be challenged, threatened, both or neither.

The TCTSA (Jones et al., 2009) further expanded on the BPSM by first clarifying the cognitive appraisal process that influences an athlete entering a challenge or threat state. Outlining the influence of self-efficacy beliefs, perceived control, and achievement goals on determining CAT states in athletes, the model highlights how the sources of self-efficacy (performance accomplishments, vicarious experiences, verbal persuasion, and physiological states), as proposed by Bandura (1986), contribute to the belief athletes may have in their ability to cope with the demands of a situation. The TCTSA suggests that a challenge state is more likely to be experienced if an athlete has high self-efficacy, a high perception of control and typically adopts approach goals. In contrast, athletes will more likely experience a threat state if they have low self-efficacy, low perception of control and are more likely to adopt avoidance goals. The TCTSA also states that the three constructs are all interrelated and that all three constructs are required for a challenge state.

The TCTSA incorporates the physiological responses as proposed within the BPSM, however, it offers a more detailed description of the emotional response. TCTSA, much like the BPSM predicts that positive emotions will be typically associated with a challenge state while negative emotions will usually be associated with a threat state. However, unlike the BPSM, the TCTSA states that negative emotions (e.g., anger or anxiety) are not exclusively associated with a threat state and can, on occasion, be experienced in a challenge state; during this state, individuals are more likely to perceive these emotions as facilitative. This finding is explained as CAT states reflect motivational states, and high-intensity emotions of a negative nature can serve a motivational purpose and would, therefore, be more consistent with a challenge state (Jones et al., 2009). This is supported by research such as Jones and Uphill (2004) who stated that athletes could enter a competition feeling anxious, but they view their anxiety as likely to help performance.

Previous research investigating CAT states have suggested that individuals in the challenge state are more likely to produce a superior athletic performance than when in a threat state (e.g., Blascovich et al., 2004; Moore et al., 2012; Turner et al., 2013). A recent systematic review conducted by Hase, O’Brien et al. (2019) found that in 24 of 38 (74%) studies, a challenge state was associated with enhanced performance. One study found an effect favoring a threat state and nine studies reported no significant impact on performance. Further to this point, Vine et al. (2016) suggested that during a threat state, individuals’ attentional and visuomotor control skills become disrupted, leading them to become distracted by less relevant stimuli and suffer a decrease in performance.

Research has also suggested that, during a challenge state, athletes interpret emotions as facilitative, whereas, in a threat state, they view emotions as debilitative (Skinner & Brewer, 2004). Previous studies have adopted physiological measures such as cardias reactivity to capture challenge and threat states (e.g. Allen et al., 2012; Arthur et al., 2019; Meijen, et al., 2014). Williams et al. (2010) also found that a threat state is associated with higher levels of cognitive and somatic anxiety compared to a challenge state, highlighting that athletes are typically likely to experience increased negative emotions and less likely to interpret these as facilitative. Turner et al. (2013) explored whether cardiovascular reactivity patterns could predict batting performance in elite cricketers using a bio-impedance cardiograph integrated system, while also measuring psychological responses with various psychometrics (e.g. Sport Emotion Questionnaire; Jones et al., 2005). Their results suggested that challenge reactivity was associated with superior performance. Likewise, Dixon et al. (2019) who examined cardiovascular reactivity in professional academy soccer, suggested that challenge reactivity is associated with superior performance, but they relied on self-report measures to assess participants’ emotions.

Research examining stress and coping strategies in cricket batsmen, such as Thelwell et al. (2007), emphasized that perceptions of self, match specific issues, technique, and current playing status were some of the most pertinent stressors experienced by cricket batters. Similarly, they also revealed that general cognitive strategies, emotion-focused coping, general match strategies, and, at the crease, specific cognitive strategies were the salient coping strategies employed by cricket batsmen. Neil et al. (2016) also highlighted that athletes’ appraisals of stressors were central to the stress and emotion process, thereby eliciting emotional responses that could be detrimental to performance if not successfully managed. Nicholls and Polman (2007) conducted a systematic review of stress and coping research in sport and suggested that the transactional model of stress and coping (TMSC) was supported in 46 of 64 studies; they highlighted a significant interaction between athletes experiencing stressors and the type of coping strategy they used. For example, athletes in individual sports adopted more coping strategies than did team athletes, and there was some evidence to suggest that males adopted more problem-focused coping strategies in response to stressors, while females reported using more emotion-focused coping strategies. Furthermore, previous stress and coping research in sport has often used the TMSC as a guiding framework to examine, for example, sources of stress encountered by performers (Arnold et al., 2013; Fletcher & Hanton, 2003), and coping responses to stressors (Didymus & Fletcher, 2012; Holt & Hogg, 2002).

Results from previous CAT studies underpinned by the TCTSA and BPSM highlight the advantages of collecting physiological data related to challenge and threat states, such as being able to accurately measure HR, CO and TPR. However, a limitation of previous CAT studies is they have often measured psychological responses (e.g. emotions, self-efficacy) using retrospective methods; similarly, previous stress and coping research has relied on retrospective data collection such as through interviews and self-report measures. Such retrospective data collection is subject to memory decay (Ericsson & Simon, 1993; Nicholls & Polman, 2008) and recall bias (Bahrick et al., 1996). While previous research has provided key findings, such as that challenge states were associated with superior performance and stress and coping occurred as a dynamic process during performance, the present study, aimed to further develop the stress and coping literature by using the BPSM and TCTSA as guiding frameworks. Likewise, this study extended previous research by examining psychological responses, specifically the stressors and coping responses of cricket batsman, as they occurred live in the moment. These methods were intended to reduce retrospective recall and prevent the loss of vital information through memory decay (Ericsson & Simon, 1993; Nicholls & Polman, 2008), while also enhancing confidence in the accuracy of athletes’ psychological responses during challenge and threat states.

Think Aloud (TA) offers opportunities for researchers to capture and examine thought processes during the performance of a task (Ericsson & Simon, 1980). Ericsson and Simon (1993) proposed three levels to verbally reporting data. Level 1 involves participants vocalizing inner speech without any effort to communicate their thoughts. Level 2 requires participants to vocalize inner speech and internal representations that are not initially part of inner speech (e.g., sensory experiences, feelings, movements). Level 3 requires participants to expand on merely verbalizing inner speech by explaining thoughts and motives. In line with the majority of TA sport psychology research, participants in the present study were required to engage in Level 2 verbalizations. Level 2 was chosen as it provides access to information from an individual’s short term memory (STM; Eccles, 2012), and participants are not required to further explain their motives, which, given the requirements of the task, participants may have struggled to do.

Recently, researchers have used TA to investigate sport psychology phenomena. For example, Swettenham et al. (2020) investigated stress and coping during practice and competitive conditions and examined gender differences across conditions using Level 2 TA Results suggested that males verbalized significantly more stressors related to performance during the competition condition and more physical stressors during the practice condition, whereas females more frequently verbalized external stressors. Whitehead et al. (2016), adopted Level 2 TA and also found that higher-skilled golfers made significantly more verbalizations per shot compared to lower-skilled golfers. Similarly, when under pressure, higher-skilled golfers shifted cognition and verbalized significantly more technical aspects of motor control, consistent with Masters’s (1992) reinvestment theory. Kaiseler et al. (2013) examined gender differences in stress, appraisals and coping during a golf putting task, and their results highlighted both significant differences in the frequency of stressors verbalized between genders and significant differences in performance appraisals between genders when participants were in identical achievement situations. These studies provide evidence for the suitability of TA as a method for collecting data related to the frequency of verbalized stressors and coping strategies during threat and challenge states. Similarly, previous TA research also highlighted how qualitative data can be coded quantitatively as, for example, by coding the frequency of verbalized stressors.

Potential limitations of adopting TA methodology include the process of requiring TA from participants during a task, as this may interfere with task performance. Whitehead et al. (2015, 2016) addressed these concerns by investigating the effects of Level 2 and Level 3 verbalizations on the performance of skilled golfers. Results indicated that neither level of verbalizations significantly impacted task performance. Similarly, a meta-analysis conducted by Fox et al. (2011) suggested that verbalizations during performance of cognitive tasks had no impact on performance and, in fact, participants who were instructed to explain their thoughts (Level 3 verbalization) improved their performance. While research suggests Level 3 TA has no significant impact on cognitive tasks, the complexity of the present task led to the decision that Level 2 TA would provide sufficient data without influencing task performance.

Thus, in the present study, we aimed to use TA to expand on previous research by investigating stress and coping of young cricket batters during CAT states. Underpinned by the BPSM, TCTSA and previous research (e.g. Moore et al., 2013; Thelwell et al., 2007; Turner et al., 2013; Whitehead et al., 2016) we predicted that participants would verbalize significantly more stressors during the threat condition compared to the challenge condition. Likewise, we hypothesized that there would be no significant difference in the total number of verbalizations made in relation to coping strategies between the threat and challenge condition. Finally, in line with Masters (1992) reinvestment theory which predicts that, under pressure, athletes verbalize more technical elements of motor control, we hypothesized that participants would make more technical verbalizations during the threat condition compared to the challenge condition.

## Method

### Participants

Ten male elite-level junior cricket batsman aged 16-17 years participated in the present study. This sample size was based on previous similar research (e.g., Samson et al., 2017; Whitehead et al., 2018). Participants were recruited from a County Cricket Boards’ excellence training program. The excellence program represents the last training stage for athletes before coaches select their squad for the forthcoming cricket season. We adopted a within-subject design whereby all participants took part in both threat and challenge conditions. Participants were recruited using a purposeful sampling technique, whereby the lead researcher, who also acted as a trainee sport and exercise psychologist for the County Cricket Board, identified eligible participants currently enrolled in the excellence program (so as to ensure high level athletic skills) who would provide insightful information that would answer the research question (Patton, 2002). To prevent demand characteristics such as verbalizing the thoughts participants believed their coaches might want to hear, we informed participants that the coaching staff would not hear their recordings.

### Equipment

Participants completed each task with their cricket equipment (e.g., cricket bat, cricket pads, cricket helmet, cricket gloves, etc.) in an indoor training venue, batting into a training cricket net. A bowling machine delivered the balls to ensure consistency in speed and location of delivery across participants. To record verbalizations during tasks, a recording device was placed in the pocket of the participant, and a wire running inside participants’ shirts connecting the microphone to the recording device was clipped onto the collar.

### Procedure

Once ethical approval for the study was acquired from the overseeing ethics committee, the performance director for the county cricket board was approached and provided with a research information sheet. The aims of the research and the requirements of the athlete’s participation were explained, and we then obtained the director’s consent to approach athletes. Participant athletes who met the initial eligibility criteria attended an optional workshop to provide a brief of the research aims, and participants who expressed an interest in participating were supplied with an information sheet. When the number of participants required for the study had been satisfied, we obtained parental consent from each participant, and participants took part in TA training exercises. We briefed participants on TA and informed them that they would be required to verbalize what they were thinking (Level 2 TA; Ericsson & Kirk, 2001). Participants then took part in a series of TA practice tasks, as per the recommendations of previous TA literature (Eccles, 2012). Tasks included: (a) counting the number of dots on a page, (b) a problem-solving task, and (c) an arithmetic task. Following training, participants then had a practice session, batting in the cricket nets to ensure they felt comfortable performing the task while wearing the equipment. Participants were also required to verbalize during this session as this also presented an ideal opportunity for the researcher to provide the participant some feedback regarding TA directly related to the experimental task, and for the participant to ask any questions regarding the use of TA if they were unsure. For example, if participants were not verbalizing enough, or finding difficulty in verbalizing during the task, the researcher could address this to ensure data collected during the experiment would be at a satisfactory level. Once participants felt comfortable with the procedure, they took part in the first condition, either the challenge or threat condition. To prevent any order effects and in line with the BPSM and TCTSA, which state that CAT states may be influenced by previous experience, participants randomly started with either the challenge or threat condition. For both conditions, participants were required to face 30 balls from a bowling machine and score 36 runs, with three runs added to the total each time they lost their wicket. The run demands were calculated based on previous similar research (e.g. Turner et al., 2013) and following discussions with the lead coach.

### Challenge Condition

To encourage participants in a challenge state, we provided participants with challenge instructions adapted from previous research (e.g. Moore et al., 2012; Moore et al., 2013), encouraging participants to view the task as a challenge to be met and overcome, to believe they are capable of overcoming the challenge, and affirming this message by stating that previous batsmen have completed the task comfortably. Following challenge instructions and before the start of the task, to ensure participants were in a challenge state, their demand and resource evaluations were measured using two items from the cognitive appraisal ratio (Tomaka et al., 1993). Participants were asked, “How demanding do you expect the upcoming task to be?” and “How able are you to cope with the demands of the upcoming task?” Items were measured on a 6-point Likert scale, with 1= not at all and 6= extremely. As per Moore et al. (2013) recommendations, a score was calculated by subtracting demands from resources (range of -5 to +5); positive scores reflected a challenge state, and negative scores reflected a threat state (see Tomaka et al., 1993). All participants scores reflected a challenge state (i.e., all participants gave a positive score). Participants then completed the challenge condition and were reminded to verbalize thoughts between shots and not during shots to avoid interference with motor movement during the execution of the skill (Schmidt & Wrisberg, 2004).

### Threat Condition

The second condition involved promoting participants into a threat state. Similar to the challenge condition, participants were required to face 30 balls from a bowling machine and score 36 runs, with three runs added to the total each time they lost their wicket. Participants were provided with threat instructions adapted from previous research (e.g., Moore et al., 2012; Moore et al., 2013) highlighting the difficulty of the task and that previous participants had failed to score the required number of runs. As with the challenge condition, all participants answered two items from the cognitive appraisal ratio to ensure participants were in a threat state. All participants scores reflected a threat state (i.e., all participants gave a negative score). Participants then completed the threat condition and were reminded to verbalize thoughts between shots and not during shots to avoid interference with motor movement during the execution of the skill (Schmidt & Wrisberg, 2004).

### Data Analysis and Research Credibility

In this study we adopted a post-positivist epistemology in line with much of the previous TA research (e.g., Arsal et al., 2016; Nicholls & Polman, 2008; Swettenham et al., 2020; Whitehead et al., 2019). We feel that is essential to state a paper’s philosophical position as doing so provides transparency and helps to refine and clarify the research method (Easterby-Smith et al., 2002). Following data collection, audio files were transcribed verbatim, and checks for relevance and consistency were made, achieved via immersing in the data and using a critical friend. Transcripts were subjected to line by line content analysis (Maykut & Morehouse, 1994) to identify themes in participants’ thought processes in both conditions. Similar to Kaiseler et al. (2013), verbalizations that caused the participant’s negative concern or worry or had the potential to do so were coded as stressors; and verbalizations in which participants attempted to manage a stressor, were coded as coping strategies. Initially, participant’s data were analyzed using an inductive thematic analysis. This involved the author reading and re-reading all transcripts of interviews (immersion in the data) using Nvivo 10 (step 1). Following this, the researcher developed a list of codes from the ﬁrst two transcripts. At this stage, the initial codes were reviewed and considered by a critical friend (step 2). Research (e.g., Saldana, 2013) has provided support for this collaborative approach to coding, as it allows a “dialogic exchange of ideas.” From the initial inductive process, codes were grouped into stressors and coping responses, and Lazarus and Folkman’s (1984) coping responses of emotion and problem-focused coping were used in a deductive way to allocate the initial inductive ‘coping responses’ into these coping responses. These deductive codes were then used as a point of reference to subsequently analyze the remaining transcripts. However, as new codes were identified from the data, for example, ‘gathering information,’ they were included as part of the analysis. We then were able to follow the saliency of these new codes throughout the data, adding new and different themes to those previously identified. Again this process was considered and reviewed by a critical friend. This process followed recommendations from Smith and McGannon (2018) to ensure data quality and rigor. In this way, 11 secondary themes were grouped into four primary themes for both the threat and challenge conditions (Table 1).

**Table 1.** Coding Framework Used to Analyse Verbalisations for Challenge and Threat Conditions.

Primary theme	Secondary theme	Description	Example
Emotion-focused coping	Emotional release	Verbalization made related to releasing negative emotions such as frustration and anger or expressing emotions.	“Argh, why did I try and do something stupid then?”
	Relaxation	Verbalizations made regarding remaining calm and relaxed or efforts by athlete to be in a relaxed state.	“Stay calm, come on, relax again, come on.”
	Positive self-talk	Verbalizations made regarding positive self-statements.	“Really good, good hand work, good contact with the ball, keep working”
Problem-focused coping	Technical instruction	Verbalizations made regarding technical instructions or corrections.	“Watch that ball, keep your eye on it.”
	Planning	Verbalizations made regarding tactics and planning for upcoming shots.	“Probably try and hit it through long on, that’s where the runs will come from”
	Increased effort	Verbalizations made regarding increasing effort and motivation towards task.	“Keep backing yourself come on, stick with it.”
	Concentration	Verbalizations made regarding increasing or remaining concentrated and focused.	“Just keep focussed, should be easy work from here.”
Stressors	External	Verbalizations made regarding external factors that may have a detrimental impact on athletic performance.	“It is a hard length to hit, can’t really get underneath the ball.”
	Performance	Verbalizations made regarding factors related to performance of skill.	“Not connected with that one very, struggling to find the middle of the bat here.”
	Pressure	Verbalizations made regarding factors related to feeling or experiencing pressure.	“Need some boundaries again now, pressure is building”
Gathering Information		Verbalizations made regarding gathering information from the environment or situation.	“15 runs of 30 balls, comes at around 3 an over.”

In line with most previous TA research in sport psychology (e.g., Kaiseler et al., 2013; Swettenham et al., 2020; Whitehead et al., 2016) and in keeping with the philosophical position adopted by this paper, we quantified the qualitative data by taking a similar coding framework to that used in previous research (e.g., Kaiseler et al., 2013). Each time a theme was verbalized it received a frequency count (Table 2), and these data were then statistically analyzed to determine any significant differences between frequency of verbalizations for each theme. First, we conducted an outlier analysis, and data were found to be normally distributed; then a series of parametric tests were conducted. As this study adopted a repeated measures design, we conducted a paired samples *t*-test to investigate differences between the coded themes for each condition. Similarly, we conducted a paired samples *t*-test to examine differences between demand/resource evaluation scores between threat and challenge conditions. A 95% confidence interval was used to determine the significance levels of the data *(p ≤* 0.05). Effect sizes were reported using Cohen’s (1988) threshold values: small (*d* = 0.2), medium (*d* = 0.5), and large (*d* = 0.8).

**Table 2.** Mean, Standard Deviation Values and Percentage and Total Frequency of Verbalizations for Primary and Secondary Stressors.

	Threat condition			Challenge condition		
Themes	Mean	SD	Total (%)	Mean	SD	Total (%)
Emotion-focused coping	**8.70**	**7.24**	**87 (21.9%)**	**7.7**	**3.62**	**77 (24.4%)**
Emotional release	2.70	2.26	27 (6.8%)	1.30	1.06	13 (4.1%)
Positive self-talk	4.00	2.83	40 (10.1%)	5.60	3.47	56 (17.8%)
Relaxation	2.00	4.00	20 (5.0%)	0.80	0.63	8 (2.5%)
Problem-focused coping	**14.6**	**6.77**	**146 (36.8%)**	**18.3**	**5.19**	**166 (52.5%)**
Concentration	2.10	2.38	21 (5.3%)	3.20	2.04	32 (10.1%)
Increasing effort	2.70	2.21	27 (6.8%)	4.50	3.21	45 (14.2%)
Planning	5.30	2.75	53 (13.4%)	4.20	2.62	42 (13.3%)
Technical instruction	4.50	2.42	45 (11.3%)	4.70	2.91	47 (14.9%)
Stressors	**12.20**	**4.83**	**123 (31.0%)**	**4.40**	**2.63**	**44 (13.9%)**
External	4.10	3.21	41 (10.3%)	1.70	1.50	17 (5.4%)
Performance	5.80	2.90	58 (14.6%)	2.30	2.00	23 (7.3%)
Pressure	2.40	1.17	24 (6.1%)	0.40	0.97	4 (1.2%)
Information gathering	4.10	2.77	41 (10.3%)	2.90	1.60	29 (9.2%)
Total verbalizations	**39.7**	**11.60**	**397 (100%)**	**31.6**	**8.72**	**316 (100%)**

Bold text represents the overarching primary themes of coping and stressors.

## Results

The frequency of verbalizations for each theme across each of the two conditions (threat and challenge) were analyzed using a paired samples *t-*test to test for significance, and a 95% confidence interval was applied. Effect sizes are reported using Cohen’s *d* values (δ). Table 1 presents the coding framework used by the researcher to analyze participant verbalizations. Descriptions of secondary theme characteristics and examples of raw data quotes are provided. Table 2 presents the means and standard deviations of primary and secondary themes, as well as the percentage and total frequency of verbalizations across both conditions.

### Demand/Resource Evaluation

A paired-samples *t*-test was used to determine if there was a significant difference between demand/resource evaluations made before participation in the challenge and threat condition. Effect sizes are reported using Cohen’s *d* values. Results indicated a significant difference between conditions with a large effect size. (*Threat condition: M* = −3.30, *SD* = 0.95; *Challenge condition: M* = 4.1, *SD* = 0.74; *t*(9) = −18.50, *p* = .000, δ = −0.94) . This finding highlights that challenge and threat states were successfully manipulated.

### Stressors

Secondary themes that emerged from the data related to stressors verbalized that were external stressors, performance stressors, and pressure (see Table 1 for examples). To analyze coded verbalizations made by participants in relation to stressors experienced across both conditions, a paired samples *t*-test test was conducted. Significant differences were found for total verbalizations made regarding stressors and a large effect size was reported. (*Threat condition: M* = 12.2, *SD* = 4.83; *Challenge condition*: *M* = 4.4, *SD* = 2.63; *t*(9) = 5.374, *p* = .000, δ = −1.53). Focusing specifically on types of stressors reported by participants, when in a threat state, participants significantly verbalized more about external stressors compared to when in a challenge state while a large effect size was also observed. (*Threat condition: M* = 4.1, *SD* = 3.21; *Challenge condition: M* = 1.7, *SD* = 1.49; *t*(9) = 2.571, *p* = .030, δ = 0.96). There were also significantly more verbalizations (large effect size) made by participants related to performance stressors (*Threat condition: M* = 5.8, *SD* = 2.90; *Challenge condition: M* = 2.3, *SD* = 2.00; *t*(9) = 3.612, *p* = .006, δ = 1.41). Finally, verbalizations coded as pressure stressors, (i.e., verbalizations regarding factors related to feeling or experiencing pressure) were analyzed. There was a large effect size and significant difference between the number of verbalizations made when in a threat state compared to a challenge state (*Threat condition: M* = 2.4, *SD* = 1.17; *Challenge condition: M* = 0.40, *SD* = 0.97; *t*(9) = 3.612, *p* = .001, δ = 1.87). These results all indicate that when in a threat state, there is a significant main effect with participants experiencing and verbalizing more stressors than when in a challenge state. These findings offer support to the first hypothesis and provide further explanations as to why performance is more likely to decrease when in a threat state compared to a challenge state, since an increased number of reported stressors indicates more instances when the participant has experienced and reported verbalizations that have caused either negative concern or worry.

### Emotion-Focused Coping

Secondary themes that emerged from the data related to emotion-focused coping were emotional release, relaxation, and positive self-talk (see Table 2 for examples). A paired samples *t*-test was carried out on the total number of verbalizations for the coded data related to emotion-focused coping. There were no significant differences between any of the secondary themes related to emotion-focussed coping. Total emotion-focused verbalizations for threat and challenge conditions were not significantly different and demonstrated a small effect size (*Threat condition: M* = 8.70, *SD* = 7.24; *Challenge condition: M* = 7.70, *SD* = 3.62; *t*(9) = .525, *p* = .612, δ = 0.18). Emotional release verbalizations between threat and challenge conditions were also not significantly different and demonstrated a medium effect size (*Threat condition: M* = 2.70, *SD* = 2.26; *Challenge condition: M* = 1.30, *SD* = 1.16; *t*(9) = 2.14, *p* = .061, δ = 0.78). Similarly, a small effect size with no significant differences were found between threat and challenge conditions for relaxation (*Threat condition: M* = 2.00, *SD* = 4.00; *Challenge condition: M* = 0.80, *SD* = 0.63; *t*(9) = .970, *p* = .357, δ = 0.42). Finally, no significant differences were identified between conditions for positive self-talk while a medium effect size was reported (*Threat condition: M* = 4.00, *SD* = 2.83; *Challenge condition: M* = 5.60, *SD* = 3.47; *t*(9) = −1.99, *p* = .078, δ = −0.51). These results suggest that participants did not verbalize more emotion-focused coping strategies when in a challenge or threat state. This finding provides support for this study’s second hypothesis.

### Problem-Focused Coping

Secondary themes that emerged from the data related to problem-focused coping were technical instruction, planning, increasing effort, and concentration (see Table 1 for examples). A paired samples *t*-test was carried out on verbalizations for the coded data related to problem-focused coping. First, total number of verbalizations made by participants related to problem-focused coping strategies was analyzed, and no significant differences were found between the threat and challenge condition (large effect size) (*Threat condition: M* = 14.6, *SD* = 6.77; *Challenge condition: M* = 18.3, *SD* = 2.19; *t*(9) = −1.713, *p* = .121, δ = −1.90) . Analyzing secondary themes, there were no significant differences for total number of verbalizations coded related to concentration between the threat condition (medium effect size) (*Threat condition: M* = 2.10, *SD* = 2.38; *Challenge condition: M* = 3.20, *SD* = 2.04; *t*(9) = −1.295, *p* = .227, δ = −0.50). No significant differences were identified for verbalizations regarding increasing effort condition (medium effect size) (*Threat condition: M* = 2.70, *SD* = 2.21; *Challenge condition: M* = 4.50, *SD* = 3.21; *t*(9) = −1.575, *p* = .150, δ = −0.70). Verbalizations made in relation to planning demonstrated a small effect size and were not found to be significantly different (*Threat condition: M* = 5.3, *SD* = 2.76; *Challenge condition: M* = 4.20, *SD* = 2.61; *t*(9) = .879, *p* = .402, δ = 0.41). Finally, there was no significant difference and a small effect size for verbalizations made in relation to technical instruction between threat and challenge conditions (*Threat condition: M* = 4.5, *SD* = 2.42; *Challenge condition: M* = 4.70, *SD* = 2.91; *t*(9) = −1.43, *p* = .889, δ = −0.07). Thus, participants did not verbalize more problem-focused coping strategies when in a challenge or threat state, supporting this study’s second hypothesis. As there were also no significant differences between the two conditions for technical verbalizations, there was also support for the third hypothesis.

### Gathering Information

Verbalizations made in relation to gathering information were statements made in relation to obtaining information from the environment or situation to facilitate performance. A paired-samples *t*-test was conducted on verbalizations related to gathering information, and no significant differences were found (medium effect size) (*Threat condition: M* = 4.10, *SD* = 2.77; *Challenge condition: M* = 2.90, *SD* = 1.59; *t*(9) = 1.450, *p* = .181, δ = 0.53).

### Total Verbalizations

Mean, standard deviation values, and total verbalizations and percentages of primary and secondary theme verbalisations are presented in Table 2. A paired-samples *t*-test was performed on the total number of verbalizations across both conditions. No significant differences were found (medium effect size) (*Threat condition: M* = 39.70, *SD* = 11.60; *Challenge condition: M* = 31.6, *SD* = 8.72; *t*(9) = 1.727, *p* = .118, δ = 0.79).

## Discussion

In the present study we aimed to investigate stress and coping of academy cricket batsmen during CAT states using Level 2 TA. First, results indicated a significant difference for demand and resource evaluation scores taken prior to participation in the threat and challenge conditions, meaning that participants were in a challenge state for the challenge condition and in a threat state for the threat condition. Results supported the first hypothesis, predicting that participants would significantly verbalize more stress sources during a threat state compared to a challenge state. Results also supported the second hypothesis, predicting that there would be no significant difference in the number of verbalizations made concerning coping strategies between challenge and threat conditions. Results did not provide support for the third hypothesis that participants would make more technical verbalizations during a threat state compared to a challenge state, as there were no significant differences in technical verbalizations. Finally, results indicated no significant differences in the total number of verbalizations made in relation to gathering information between the two conditions.

There were significant differences found between total overall verbalizations for stressors experienced by participants between both conditions. Significant differences were also found for each primary stressor theme (external, performance, and pressure stressors). These findings provide further support for both the BPSM and TCTSA and further extend the scope to where this knowledge can be applied. The results suggested that when in a threat state, participants are more likely to experience stress sources than when in a challenge state. Both models suggest that if athletes appraise that they do not possess the coping resources required to manage a situation, they will enter a threat state. This finding is in line with Moore et al. (2013) who suggested demand/resource evaluations made before a competition can significantly predict competitive performance. When participants evaluated the competitive demands to outweigh their resources (i.e., a threat state), this was significantly associated with reduced performance compared to perceiving their resources to match or exceed the competitive demands (i.e., a challenge state).

Previous research investigating stress in sport suggested that athletes experience a wide variety of stressors, similar to those identified in the present study (external stressors, performance stressors, and pressure). For example, Swettenham et al. (2020) highlighted external stressors as a salient stressor in tennis players. The findings from the present study extend this by highlighting that external stressors are more likely to be reported during a threat state than a challenge state. Similarly, the findings from the present study support previous research investigating stress sources in cricket batsman. Thelwell et al. (2007) suggested cricket batsman experience a wide variety of stressors when performing in competition, and a few examples include perceptions of self, match specific issues and technique. In the current study, performance-related stressors were the most frequently cited stressors across both conditions. However, performance-related stressors were reported significantly more often by participants when in a threat state compared to a challenge state. This finding suggests that during a threat state, participants more frequently verbalize stressors related to skill performance, probably because participants’ performances decline while in a threat state. Of the ten participants, only one participant in a threat state successfully completed the task (i.e. scored the target amount of runs), whereas all participants in a challenging state were successful. This provides further support for previous research (e.g., Blascovich et al., 2004; Moore et al., 2012; Turner et al., 2012). Hase, O’Brien et al. (2019) systematic review suggested that a challenge state is beneficial to performance. The findings from the present study extend the work in previous research by highlighting that, in real-time, participants in a threat state (versus a challenge state) verbalize significantly more stressors. This finding offers a potential explanation for why athletic performance is more likely to decrease when athletes are in a threat state.

Despite the significant increase in stressor verbalizations made during a threat state, there was no significant difference found in the number of verbalizations made to cope with stressors reported by participants (external stressors, performance stressors, and pressure). This finding suggests that athletes in a threat state will experience more stressors without verbalizing significantly more coping strategies. The BPSM and TCTSA propose that during a threat state athletes have appraised that task demands outweigh their resources; therefore, this finding enhances our confidence in previous research. Perhaps surprisingly, this study’s results also indicated that, during a challenge state, participants did not verbalize a higher number of coping strategies. Arguably, this finding may result from some coping strategies having not been verbalized (e.g. breathing techniques). Likewise, a possible explanation for this finding may be that, during a challenge state, a higher quality of coping strategies leads athletes to naturally engage in fewer verbalizations. An alternative explanation for these findings could offer support to the ESACT (Uphill et al., 2019), suggesting that individuals can be experiencing challenges, threats, neither or both. It could be argued that this finding provides support for this model as the lack of verbalized coping responses may result from athletes being *both* challenged and threatened, rather than alternatively challenged *or* threatened (as is implied by a theory that challenge and threat are on a bipolar continuum).

The present study and previous research (e.g., Blascovich et al., 2004; Moore et al., 2012; Turner et al., 2012) highlighted how a threat state is associated with decreased performance. A potential solution to promoting a challenge state and facilitating performance may be to develop coping strategies to manage the increase in stressors. A recent paper conducted by Hase, Hood et al. (2019) specifically highlighted the potential for motivational self-talk to be used as a tool for promoting a challenge state and improving performance. Therefore, future research could further examine the effectiveness of psychological skills training, arousal reappraisal, and imagery interventions. These interventions are aimed at developing coping strategies to manage increased stressors when in a threat state; such interventions may reduce the impact a threat state may have on performance by better regulating emotional arousal and eliminating stressors.

While it was predicted that participants in the threat state would make more technical verbalizations compared to when in a challenge state, there were no significant technical verbalization differences found in this study, in contrast with previous research. For example, Whitehead et al. (2016) highlighted that higher-skilled golfers, when under pressure, were more likely to verbalize technical rules, consistent with Masters (1992) reinvestment theory. Reinvestment theory states that a skilled performer may regress to an earlier stage of learning during a stressful situation – a phenomenon referred to as choking in which there is a breakdown in performance under situations of stress or pressure (Beilock & Gray, 2012). Similarly, Vine et al. (2016) argued that during a threat state, performers are more likely to focus their attention inwardly towards internal cues. In the present study, while there were no significant differences between groups during both conditions, technical verbalizations during both conditions (11.3% and 14.9%, respectively) represented an important percentage of total verbalizations. It may be argued that this finding was due to these participants’ younger stage of development (i.e., junior athletes). At these younger ages, technical verbalizations might still be a vital training tool for athletic development, meaning that they facilitate, rather than hinder performance. For example, athletes in this study, used statements such as *“watch the ball, keep your eye on it,” “keep your feet moving”* and *“play the ball straight,”* perhaps to reinforce correct technical elements of batting. Thus, rather than hinder performance by directing attention inwardly, these verbalizations may be facilitating performance by strengthening best practice. In this way, they may be a useful coping technique for athletes at this stage of development. Further research is needed, however, to better understand the underlying mechanisms for this finding.

## Limitations and Future Research

A potential limitation of the present study is the lack of any physiological participant measures during CAT states. The present study relied on self-report measures, including two items from the cognitive appraisal ratio (Tomaka et al., 1993), to determine whether participants were in a challenge or threat state. Previous research has used alternative measurement methods, such as Turner et al. (2012), who measured CV reactivity and self-report measures of self-efficacy, control, achievement-goals, and emotions. Similarly, Moore et al. (2013) used cardiovascular measures, performance measures, and a series of self-report measures. While physiological testing would not have further addressed the present studies main aims, they may have contributed to a determination of the participants’ CAT states, increasing the validity and reliability of obtained outcome data. Future research could, therefore, consider this limitation and better address it. Level 2 TA does not require participants to expand on their thoughts or provide motives/explanations for verbalizations, and this may have limited data in this study. However, we felt that, given the dynamic nature of batting in cricket, Level 2 TA provided sufficient data while limiting potential batting performance disruptions.

Future research might examine the effectiveness of interventions aimed at promoting athletes’ challenge state and preventing their threat state. Based on the results of the present study, such interventions should focus on developing coping strategies to manage the increase of stressors during a threat state. Our results also suggest that stressors and the threat state had a detrimental effect on sporting performance. Hase, Hood et al. (2019) offer a potential intervention for addressing such issues (e.g., use of motivational self-talk), although the effectiveness of other psychological interventions should also be examined. Based on the findings of the present study, future research could explicitly investigate the performance impact of technical instruction in junior athletes.

## Conclusions

To conclude, in this study we used a novel approach to collect data from cricket batsmen during CAT states. We adopted an idiographic design, as advocated by Lazarus (2000) and extended it to previous CAT research by soley examining stress and coping during CAT states as they occurred. Our findings provide some to support both the BPSM and TCTSA by highlighting that, during threat states, participants experience an increase in stressors compared to a challenge state. However, our results did not suggest the increase in coping strategies during a challenge state that previous theories have eluded to. Alongside this, elite junior athletes verbalized technical elements of skills during both CAT states, which they may have used as a coping mechanism, although further research is needed to verify this possibility. Future research should investigate potential interventions aimed at promoting a challenge state, perhaps by helping athletes reduce the number of stressors experienced and increase coping skills matched to perceived task demands.

## Article Notes

### Author Biographies

**Michael McGreary**: is currently a Professional Doctorate student in Sport Psychology at Liverpool John Moores University. He works as a Lecturer in Sport Psychology at the University Campus North Lincolnshire (UCNL). He also works as an applied sport psychology practitioner in football and cricket. Michael’s main area of research focuses on Think Aloud in cricket and athlete transitions.

**Martin Eubank**: is a Principal Lecturer, Subject Head and Programme Leader for the Professoinal Doctorate in Sport and Exercise Psychology at Liverpool John Moores University. Dr Eubank is a HCPC registered Sport and Exercise Psychologist and his research focuses on sport and exercise psychology practitioner development and practice.

**Robert Morris**: is currently a lecturer in sport psychology at the University of Stirling, teaching on undergraduate and postgraduate degree programmes in sport psychology and conducting research in the areas of talent development and transitions in sport. Robert is a BASES Accredited, BPS Chartered, and HCPC Registered Sport and Exercise Psychologist and is a Fellow of the Higher Education Academy and Associate Fellow of the BPS. Robert is also an applied practitioner in sport psychology, providing support to a range of team, national, international, and Olympic athletes in sports including football, rugby union, canoe, swimming, and golf.

**Amy Whitehead**: is a Reader in Sport Psychology and Coaching at Liverpool John Moores University. Her research focuses on the use of Think Aloud to understand athlete and coach cognition and how Think Aloud can be used as a reflective practice tool for coaches. Whitehead is also a BPS accredited Sport and Exercise Psychologist working as a consultant with a variety of athletes.

### ORCID iD

Amy Whitehead https://orcid.org/0000-0003-0611-364X
